# QuickStats

**Published:** 2015-04-24

**Authors:** FROM THE NATIONAL CENTER FOR HEALTH STATISTICS

**Figure:**
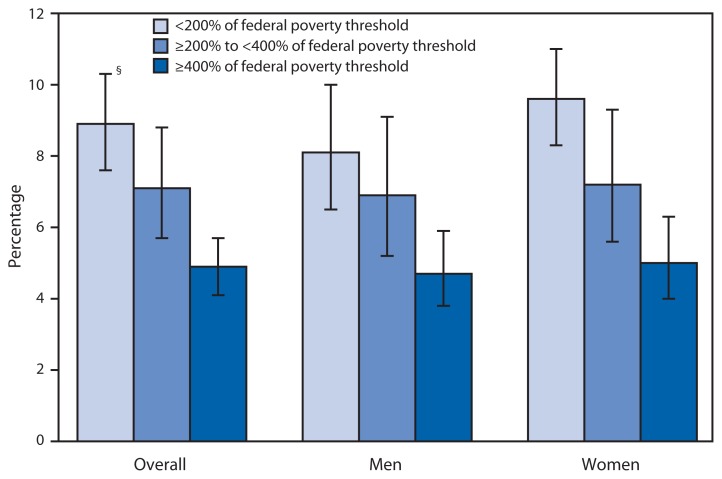
Use of Prescription Opioid Analgesics* in the Preceding 30 Days Among Adults Aged ≥20 Years, by Poverty Level^†^ and Sex — National Health and Nutrition Examination Survey, United States, 2007–2012 * During the household interview, respondents were asked, “In the past 30 days, have you used or taken medication for which a prescription is needed?” Those who answered affirmatively were asked to give their prescription medication containers to the interviewer, who then recorded the exact product name from the container’s label. Opioid analgesics are commonly prescribed for treating pain caused by surgery, injury, or health conditions such as cancer. Common opioid analgesics include hydrocodone, oxycodone, and methadone. ^†^ Poverty level was based on the family income to poverty ratio, which is the ratio of family income to the poverty threshold after accounting for inflation and family size. A ratio of 1.00 was considered representative of a poverty level at 100% of the federal poverty guideline. ^§^ 95% confidence interval.

During 2007–2012, use of opioid analgesics in the United States decreased with increasing income; 8.9% of adults aged ≥20 years who had family incomes <200% of the federal poverty threshold used an opioid analgesic in the preceding 30 days, compared with 7.1% of those with incomes 200%–399% of the poverty threshold and 4.9% of those with incomes ≥400% of the poverty threshold. The relationship between income and opioid use was observed for both men and women. Within each of the family income categories, there were no significant differences in opioid analgesic use between men and women.

**Source:** Frenk SM, Porter KS, Paulozzi LJ. Prescription opioid analgesic use among adults: United States, 1999–2012. NCHS data brief no. 189; 2015. Available at http://www.cdc.gov/nchs/data/databriefs/db189.htm.

**Reported by:** Steven M. Frenk, PhD, sfrenk@cdc.gov, 301-458-4096; Kathryn S. Porter, MD, Leonard J. Paulozzi, M.D.

